# Hemoglobin and clinical outcomes of in-hospital patients with severe acute exacerbation of chronic obstructive pulmonary disease: a multicenter cohort study

**DOI:** 10.3389/fmed.2025.1674268

**Published:** 2025-10-16

**Authors:** Suji Wu, Qun Yi, Yuanming Luo, Hailong Wei, Huiqing Ge, Huiguo Liu, Xianhua Li, Jianchu Zhang, Pinhua Pan, Mengqiu Yi, Lina Cheng, Hui Zhou, Yongjiang Tang, Haixia Zhou

**Affiliations:** ^1^Department of Respiratory and Critical Care Medicine, West China Hospital, Sichuan University, Chengdu, Sichuan, China; ^2^Sichuan Cancer Hospital and Institution, Sichuan Cancer Center, Cancer Hospital Affiliated to School of Medicine, UESTC, Chengdu, Sichuan, China; ^3^State Key Laboratory of Respiratory Disease, Guangzhou Medical University, Guangzhou, Guangdong, China; ^4^Department of Respiratory and Critical Care Medicine, People’s Hospital of Leshan, Leshan, Sichuan, China; ^5^Department of Respiratory and Critical Care Medicine, Sir Run Run Shaw Hospital, Zhejiang University School of Medicine, Hangzhou, Zhejiang, China; ^6^Department of Respiratory and Critical Care Medicine, Tongji Hospital, Tongji Medical College, Huazhong University of Science and Technology, Wuhan, Hubei, China; ^7^Department of Respiratory and Critical Care Medicine, The First People’s Hospital of Neijiang City, Neijiang, Sichuan, China; ^8^Department of Respiratory and Critical Care Medicine, Union Hospital, Tongji Medical College, Huazhong University of Science and Technology, Wuhan, Hubei, China; ^9^Department of Respiratory and Critical Care Medicine, Xiangya Hospital, Central South University, Changsha, Hunan, China; ^10^Department of Emergency, The First People’s Hospital of Jiujiang, Jiujiang, Jiangxi, China; ^11^Department of Respiratory and Critical Care Medicine, The Affiliated Hospital of Chengdu University, Chengdu, China

**Keywords:** anemia, polycythemia, adverse outcome, acute exacerbation, chronic obstructive pulmonary disease

## Abstract

**Background:**

Hemoglobin is one of the most common laboratory tests for hospitalized patients, and both anemia and polycythemia are common comorbidities in severe acute exacerbation of chronic obstructive pulmonary disease (AECOPD). However, limited evidence focuses on the predictive value of anemia or polycythemia for in-hospital adverse outcomes of severe AECOPD.

**Methods:**

The patients hospitalized for severe AECOPD were prospectively enrolled from 10 medical centers in China. They were categorized into three groups: anemia, normal, and polycythemia, based on their hemoglobin levels on-admission. The adverse outcomes which included all-cause in-hospital mortality, invasive ventilation, and intensive care unit (ICU) admission.

**Results:**

A total of 9,660 AECOPD inpatients were included. The cohort identified a significant association between anemia and adverse outcomes when compared to the normal group (5.20% vs. 2.80%, *p* < 0.001), including In-hospital mortality (1.12% vs. 0.29%, *p* < 0.001), invasive ventilation (2.12% vs. 1.19%, *p* = 0.001), ICU admission (4.24% vs. 2.41%, *p* < 0.001). When hemoglobin was further categorized from <6 g/dL to ≥20 g/dL, and 12 to <16 g/dL was taken as reference, ORs for adverse outcomes increased with decreased hemoglobin in the overall cohort, hemoglobin<60 g/dL (OR = 7.714, 95% CI: 2.622 ~ 20.887), hemoglobin 6 to <9 g/dL (OR = 3.284, 95% CI: 2.142 ~ 4.93). Conversely, no significant relationship was observed between polycythemia and adverse outcomes when compared to the normal group. Additionally, compared with normal group, participants with anemia were found to be older and showed elevated levels of WBC, Neutrophil ratio, PCT, CRP, serum G test positive rate, GM test positive rate, BUN, creatinine and D-dimer.

**Conclusion:**

While there is no effect of polycythemia on adverse outcomes in severe AECOPD inpatients, anemia on-admission, particularly <9 g/dL, is associated with a heightened risk of adverse outcomes, which may serve as an effective biomarker of poor prognosis among inpatients with severe AECOPD.

## Background

Chronic Obstructive Pulmonary Disease (COPD) is now one of the top three causes of death worldwide and 90% of these deaths occur in low- and middle-income countries (LMICs). Based on BOLD and other large scale epidemiological studies, it is estimated that the global prevalence of COPD is 10.3%. In China, COPD was accounted for a quarter of the global COPD population. From 1990 to 2019, the incidence and prevalence numbers of COPD increased by 61.2 and 67.8%. With the increasing prevalence of smoking in LMICs, and aging populations in high-income countries, the prevalence of COPD is expected to rise (1–3). The global burden of COPD is projected to rise in the coming decades due to persistent exposure to risk factors and population aging, COPD exacerbations account for the greatest proportion of the total COPD burden on the healthcare system (4, 5). Although a major public health challenge, COPD is both preventable and treatable. Annually, numerous patients are hospitalized or die from acute exacerbation of COPD (AECOPD). In order to improve adverse outcomes, it is critical to investigate comorbidities and prognostic factors in patients with AECOPD.

Clinical studies have identified multiple mortality risk factors in AECOPD, including comorbid heart failure, pulmonary consolidation, acidosis, eosinopenia, elevated D-dimer, increased blood urea nitrogen (BUN), low body mass index (BMI), and low diastolic blood pressure (6–11). Hemoglobin, a routine laboratory parameter for hospitalized patients, is often abnormal in AECOPD, anemia and polycythemia are both common. Polycythemia may correlate with pulmonary hypertension (12, 13), venous thromboembolism (14), and even mortality (15, 16). Anemia in stable COPD patients have also been confirmed to be associated with older, more cardiometabolic comorbidities, worse dyspnea, poorer quality of life, severe airflow obstruction, reduced exercise capacity, higher exacerbation risk, and increased mortality (17–19). Severe AECOPD carries a higher risk of mortality and healthcare resource utilization, but the evidence regarding the predictive value of adverse outcomes causing by anemia or polycythemia in hospitalized patients with severe AECOPD is limited. To elucidate the clinical implications is essential for improving severe AECOPD management and outcomes. Therefore, this study is dedicated to clarify the relationship between different ranges of hemoglobin and adverse outcomes in severe AECOPD inpatients.

## Materials and methods

### Study participants and design

MAGNET AECOPD (MAnaGement aNd advErse ouTcomes in inpatients with acute exacerbation of COPD) is a prospective observational study conducted across 10 clinical centers in China. It enrolled patients hospitalized for AECOPD in large tertiary general hospitals from September 2017 to July 2021. The primary objectives were to examine the management and adverse outcomes of AECOPD inpatients and to develop early warning models for these outcomes. This sub study specifically investigated the association between admission hemoglobin levels and in-hospital adverse outcomes. This study received approval from the institutional review boards of all participating academic medical centers.

The diagnosis of AECOPD was consistent across all 10 participating medical centers and required: a history of COPD, as defined by the Global Initiative for Chronic Obstructive Lung Disease (GOLD) criteria, and patients admitted to hospitals were defined as severe AECOPD. An acute worsening of respiratory symptoms necessitating additional treatment. The exclusion criteria were as follows: (1) patients aged < 40 years; (2) patients with missing hemoglobin data; (3) patients with any of the following diseases: hematopoietic system diseases, connective tissue disease, hepatic cirrhosis, chronic kidney disease, sepsis, pneumonia, bleeding history in 1 month before admission; The admission and treatment of patients were determined by the attending physician, and no additional direct intervention was performed.

### Data collection and definitions

Sociodemographic information, ethnic group (minority or not), smoking status, BMI (weight divided by the square of height), and general vital signs including systolic blood pressure (SBP), diastolic blood pressure (DBP), pulse, as well as respiratory rate (RR) were collected immediately upon admission. The comorbidities were also recorded in detail, hypertension, coronary heart disease (CHD), chronic heart failure (CHF), Cor pulmonale, obstructive sleep apnea (OSA), and diabetes were the most common comorbidities. Laboratory data, white blood cell (WBC), hemoglobin, neutrophil ratio, platelet, eosinophil ratio, procalcitonin (PCT), C-reactive protein (CRP), 1,3-β-d glucan test (G test), galactomannan test (GM test), blood urea nitrogen (BUN), creatinine, albumin, N-terminal pro-brain natriuretic peptide (NT-proBNP), D-dimer, were also collected subsequently. Blood gas analysis was also conducted to obtain the potential of hydrogen (PH), partial pressure of oxygen in arterial blood (PaO_2_), partial pressure of carbon dioxide in arterial blood (PaCO_2_), and lactic acid (LAC). Anemia was defined as peripheral blood hemoglobin concentration <11 g/dL in females, and <12 g/dL in males, while polycythemia was defined as hemoglobin ≥15 g/dL in females, and ≥16 g/dL in males. Patients were divided into three groups based on their hemoglobin levels: anemia, normal (11–14.9 g/dL in females, 12–15.9 g/dL in males). We firstly compare the differences in comorbidities and laboratory indicators among the three groups, and further to explore the relationships between hemoglobin level and adverse outcomes of AECOPD inpatients.

### Adverse outcomes

The adverse outcomes included all cause in-hospital mortality, ICU admission, and invasive mechanical ventilation of AECOPD inpatients.

### Statistical analyses

Statistical analysis of this study was conducted by R statistical software (version 4.0.5). Descriptive analysis of quantitative data was performed using mean ± standard deviation or median plus quartile interval; qualitative data was conducted using rates and proportions. Continuous variables were compared by T tests or rank sum tests; categorical variables were compared using chi-square test. Multiple imputations (20) based on 5 replications and chained equation approach were conducted to account for missing data using the Mice package in R software if the missing values were less than 20%, and variables with a missing rate of more than 20% were excluded. To further explore the association between adverse outcomes of severe AECOPD patients and hemoglobin groups, a multivariable logistic regression analysis was performed to adjust for confounding factors. Odds ratios (OR) and 95% confidence intervals (95% CI) were calculated for each patient group based on the multivariable analysis. A *p*-value < 0.05 was considered statistically significant.

## Results

### Patient characteristics

**Figure 1:**
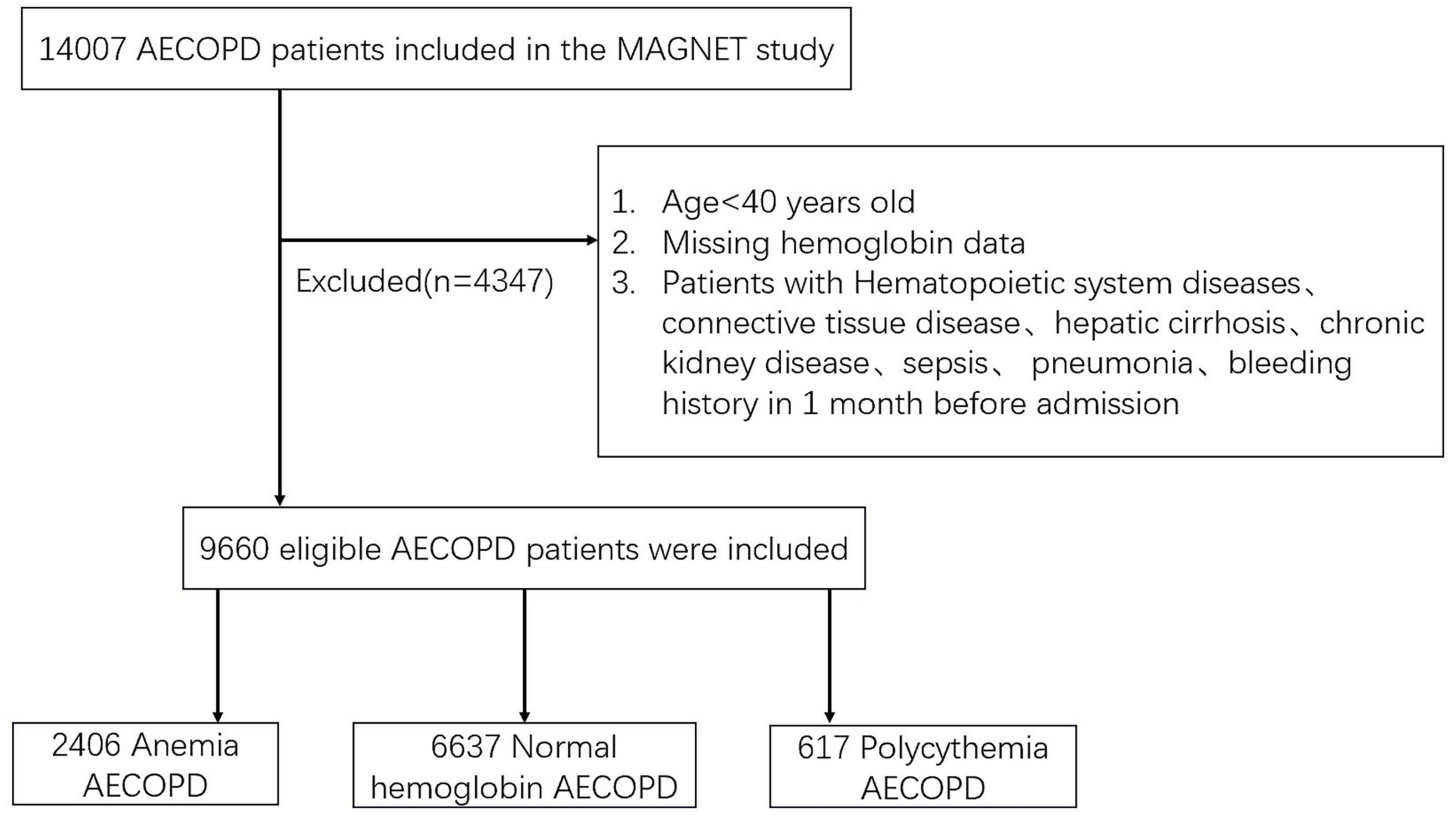
Flow chart of the study.

A total of 14,007 patients admitted for severe AECOPD were enrolled in the original registration study. Four thousand, three hundred forty-seven patients were excluded according to the exclusion criteria, and 9,660 severe AECOPD inpatients were eventually enrolled into our study. Among them, 2,406(24.91%) patients were diagnosed with anemia, 6,637(68.71%) patients had normal hemoglobin levels, and 617(6.39%) patients had polycythemia (Figure 1). Compared to the normal group, the anemia group was older (74.43 ± 9.49 vs. 71.09 ± 9.68 years, *p* < 0.001), had a lower proportion of women (15.54% vs. 20.57%, *p* < 0.001), and higher rates of hypertension (33.96% vs. 31.48%, *p* = 0.027), CHD (11.85% vs. 9.84%, *p* = 0.006), and diabetes (12.26% vs. 10.55%, *p* = 0.024), while the polycythemia group was younger (67.32 ± 10.46 vs. 71.09 ± 9.68 years, *p* < 0.001), had a higher proportion of women (24.15% vs. 20.57%, *p* = 0.041), and elevated rates of CHF (13.61% vs. 7.49%, *p* < 0.001), minority, Cor pulmonale (35.98% vs. 16.30%, *p* < 0.001), and OSA (2.11% vs. 0.60%, *p* < 0.001) (Table 1).

**Table 1:** Baseline characteristics of the study population.

Variables	Normal (*N* = 6,637)	Anemia (*N* = 2,406)	*P*^#^*-*value	Polycythemia (*N* = 617)	P^*^-value
Characteristics					
Gender (female, %)	1,365 (20.57)	374 (15.54)	**<0.001**	149 (24.15)	**0.041**
Age (years)	71.09 ± 9.68	74.43 ± 9.49	**<0.001**	67.32 ± 10.46	**<0.001**
BMI (kg/m^2^)	21.68 ± 3.01	20.97 ± 2.86	**<0.001**	22.56 ± 3.36	**<0.001**
Minority	165 (2.49)	35 (1.45)	**0.004**	59 (9.56)	**<0.001**
Smoking status			0.092		0.819
Never	2,461 (37.08)	874 (36.33)		233 (37.76)	
Current smoker	1,361 (20.51)	455 (18.91)		120 (19.45)	
Former smoker	2,815 (42.41)	1,077 (44.76)		264 (42.79)	
Comorbidities					
Hypertension	2,089 (31.48)	817 (33.96)	**0.027**	197 (31.93)	0.852
Coronary heart disease	653 (9.84)	285 (11.85)	**0.006**	44 (7.13)	**0.035**
Chronic heart failure	497 (7.49)	200 (8.31)	0.21	84 (13.61)	**<0.001**
Cor pulmonale	1,082 (16.30)	365 (15.17)	0.206	222 (35.98)	**<0.001**
OSA	40 (0.60)	5 (0.21)	**0.029**	13 (2.11)	**<0.001**
Diabetes	700 (10.55)	295 (12.26)	**0.024**	79 (12.80)	0.096
Symptoms and signs					
SBP (mmHg)	132.98 ± 18.62	131.02 ± 19.86	**<0.001**	130.69 ± 18.46	**0.003**
DBP (mmHg)	80.26 ± 11.96	76.74 ± 12.36	**<0.001**	82.53 ± 11.83	**<0.001**
Pulse (times/min)	88.92 ± 16.20	89.29 ± 17.03	0.347	88.28 ± 17.43	0.347
Respiratory rate (times/min)	20.78 ± 1.99	20.93 ± 2.21	**0.002**	20.78 ± 2.14	0.98

Data are presented as the number of patients (%), mean ± standard deviation. Those with P value < 0.05 were highlighted using the bold font. *P*^#^: The *p*-value of analysis between the anemia group and the normal hemoglobin group. P^*^: The *p*-value of analysis between the polycythemia group and the normal hemoglobin group.

BMI, body mass index; OSA, obstructive sleep apnea; SBP, systolic blood pressure; DBP, diastolic blood pressure.

Interestingly, in terms of laboratory indicators, the inflammatory markers like WBC, neutrophil ratio, PCT, CRP, and positivity rates for serum G and GM tests were higher in the anemia group compared to the normal group (*p* < 0.05). But the polycythemia group only had lower CRP (14.84 ± 28.71 vs. 19.42 ± 35.05 mg/dL, *p* = 0.002) but no significant differences in other inflammatory markers. As for blood gas, the polycythemia group had lower PaO_2_ (81.34 ± 28.95 vs. 87.85 ± 26.74 mmHg, *p* < 0.001) and higher PaCO_2_ (48.38 ± 13.23 vs. 45.47 ± 10.57 mmHg, *p* < 0.001), while the anemia group had higher PaO_2_ (92.03 ± 30.03 vs. 87.85 ± 26.74 mmHg, *p* < 0.001) (Table 2).

**Table 2:** Laboratory tests of normal, anemia, polycythemia groups.

Variables	Normal (*N* = 6,637)	Anemia (*N* = 2,406)	*P* ^#^value	Polycythemia (*N* = 617)	*P* ^*^value
WBC (10^9^/L)	8.34 ± 3.73	8.55 ± 4.46	**0.022**	8.32 ± 4.05	0.904
Neutrophil ratio (%)	72.76 ± 12.61	74.74 ± 12.69	**<0.001**	72.92 ± 12.58	0.763
Platelet (10^9^/L)	204.69 ± 80.50	231.43 ± 20.26	**<0.001**	166.96 ± 62.49	**<0.001**
Eosinophil ratio (%)	1.20(0.20,2.80)	1.0(0.10,2.80)	0.2	0.9(0.20,2.30)	**0.03**
PCT (mg/mL)	0.29 ± 3.46	0.80 ± 7.61	**<0.001**	0.16 ± 1.14	0.374
CRP (mg/dl)	19.42 ± 35.05	29.60 ± 65.05	**<0.001**	14.84 ± 28.71	**0.002**
G test (Positive, %)	103 (1.55)	65 (2.70)	**<0.001**	5 (0.81)	0.2
GM test (Positive, %)	70 (1.05)	41 (1.70)	**0.018**	6 (0.97)	1
PH	7.40 ± 0.04	7.41 ± 0.05	**<0.001**	7.39 ± 0.05	**<0.001**
PaO_2_ (mmHg)	87.85 ± 26.74	92.03 ± 30.03	**<0.001**	81.34 ± 28.95	**<0.001**
PaCO_2_ (mmHg)	45.47 ± 10.57	45.01 ± 10.94	0.067	48.38 ± 13.23	**<0.001**
LAC (mmol/L)	1.56 ± 0.47	1.55 ± 0.58	0.58	1.62 ± 0.56	**0.001**
Albumin (g/dl)	37.90 ± 4.90	34.63 ± 5.25	**<0.001**	38.40 ± 5.57	**0.015**
BUN (mmol/L)	6.15 ± 10.09	7.55 ± 22.48	**<0.001**	6.59 ± 3.61	0.28
Creatinine (μmol/L)	89.25 ± 71.92	97.62 ± 80.31	**<0.001**	98.88 ± 90.85	**0.002**
NT-proBNP (ng/dl)	4,775.91 ± 186,400.01	3,113.92 ± 45,551.26	0.665	2,709.26 ± 20,142.72	0.783
D-dimer (mg/dl FEU)	1.26 ± 11.92	1.94 ± 6.12	**0.007**	1.63 ± 8.74	0.455

Data are presented as the number of patients (%), mean ± standard deviation, or median (Q1, Q3). Those with *P*-value < 0.05 were highlighted using the bold font. *P*^#^: The *p*-value of analysis between the anemia group and the normal hemoglobin group. P^*^: The *p*-value of analysis between the polycythemia group and the normal hemoglobin group.

WBC, white blood cell; PCT, procalcitonin; CRP, C-reactive protein; G test, 1,3-β-d glucan test; GM test, galactomannan test; PH, potential of hydrogen; PaO_2,_ partial pressure of oxygen in arterial blood; PaCO_2_, partial pressure of carbon dioxide in arterial blood; LAC, lactic acid; BUN, blood urea nitrogen; NT-proBNP, N-terminal pro-brain natriuretic peptide.

**Figure 2:**
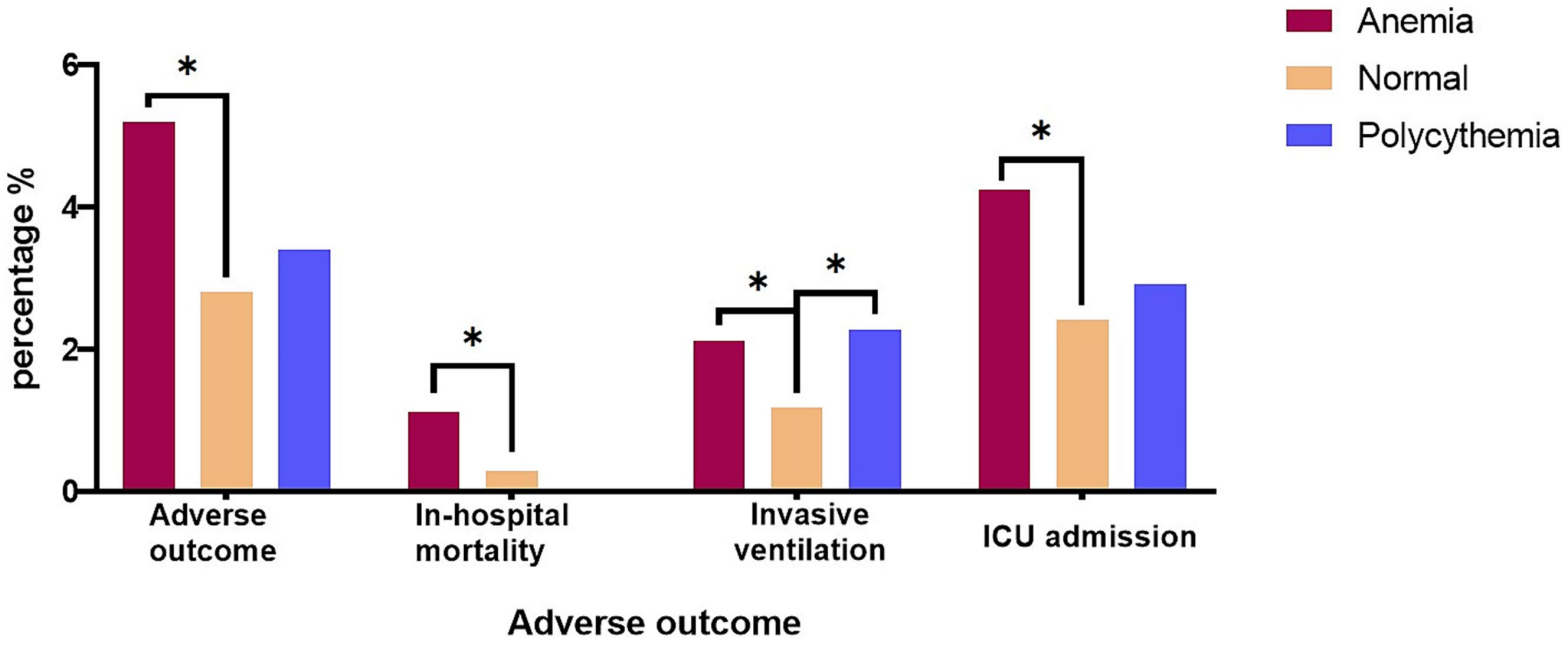
The adverse outcome among the three groups with In-hospital mortality, Invasive mechanical ventilation, and ICU admission, respectively. (*p-value < 0.05); ICU, intensive care unit.

During the hospital stay, 332(3.45%) patients suffered adverse outcomes, among them, 46 patients dead in hospital, 144 patients performed invasive ventilation, and 280 patients were admitted to the ICU. The incidence of adverse events was significantly higher in the anemia group when compared to normal group (5.20% vs. 2.80%, *p* < 0.001), no matter in terms of the in-hospital mortality, invasive ventilation, or ICU admission. The polycythemia group had a 3.40% adverse outcome rate, not significantly different from the normal group (2.80%, *p* = 0.465). Notably, despite the polycythemia group demonstrating the highest proportion of mechanical ventilation at 2.27%, no in-hospital mortality happened in polycythemia group (Table 3 and Figure 2).

**Table 3:** Adverse outcomes of normal, anemia, polycythemia groups.

Variables	Normal (*N* = 6,637)	Anemia (*N* = 2,406)	*P*^#^-value	Polycythemia (N = 617)	P^*^-value
Adverse outcome (%)	186 (2.80)	125 (5.20)	**<0.001**	21 (3.40)	0.465
In-hospital mortality (%)	19(0.29)	27(1.12)	**<0.001**	0(0.0)	0.4
Invasive ventilation (%)	79(1.19)	51(2.12)	**0.001**	14(2.27)	**0.04**
ICU admission (%)	160(2.41)	102(4.24)	**<0.001**	18(2.92)	0.5

ICU, intensive care unit. P#: The *p*-value of analysis between the anemia group and the normal hemoglobin group. P*: The p-value of analysis between the polycythemia group and the normal hemoglobin group. Those with *P* value < 0.05 were highlighted using the bold font.

The univariate and multivariate analyses revealed that hypertension, CHF, Cor pulmonale, low DBP, elevated WBC count, neutrophil ratio, serum creatinine, CRP, and PCT, decreased albumin and platelets were associated with adverse outcomes. Interestingly, anemia, but not polycythemia, showed significant correlation with adverse outcomes (*p* = 0.003) (Supplementary Tables 1, 2).

**Figure 3:**
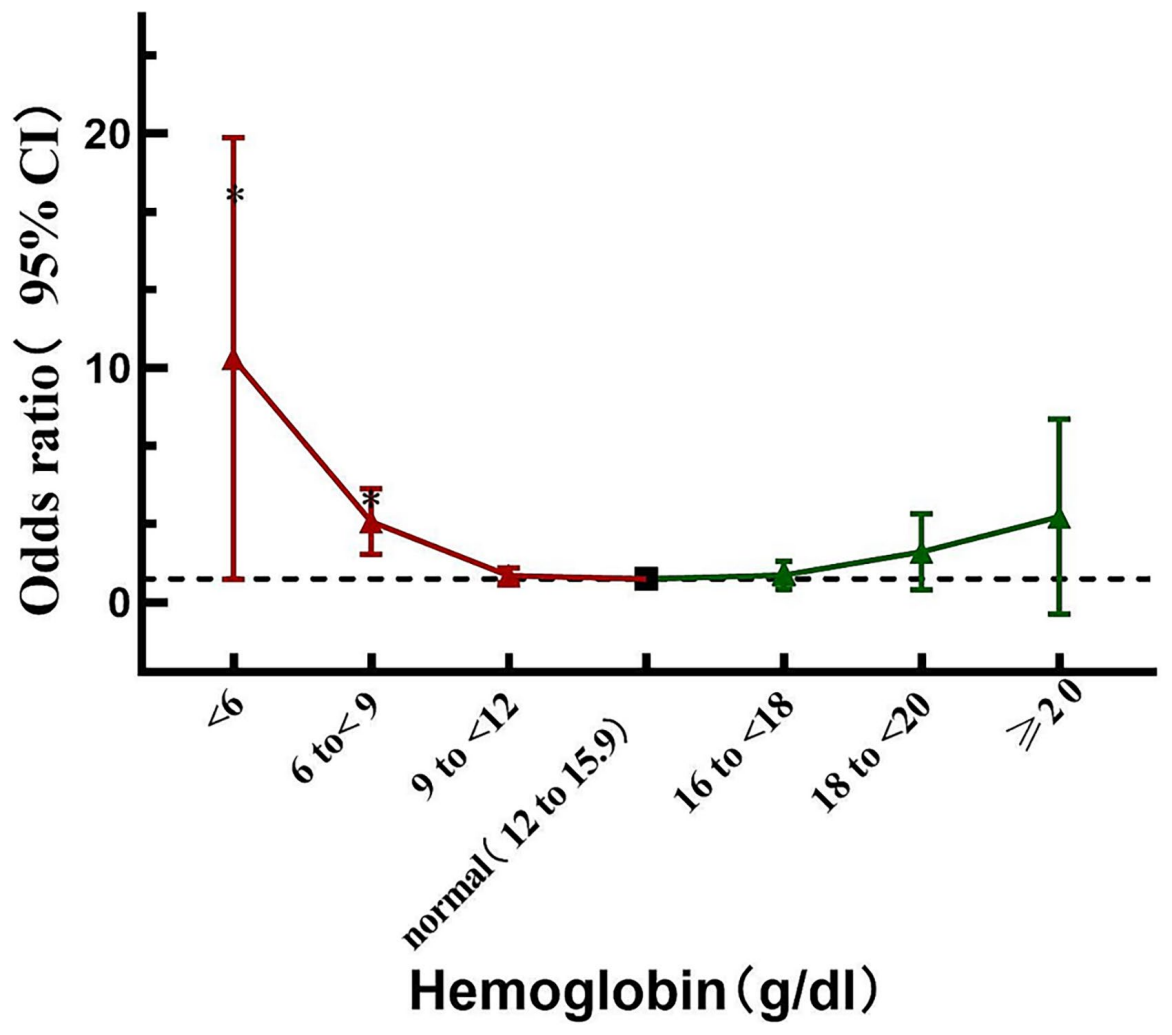
Relationship between Hemoglobin and the risk of adverse outcome; the black thick dot refers to the reference (i.e., 12 to <15.9 g/dL; the red triangle refers to the hemoglobin lower than the reference; the green triangle refers to the Hemoglobin higher than the reference range); *p < 0.05.

In order to further detect the different hemoglobin levels and the risk of adverse outcomes, stratification of hemoglobin levels from <6 g/dL to ≥20 g/dL were compared with the reference category (12–15.9 g/dL) respectively (Table 4). It is worth noting that the risk of adverse events significantly increases when hemoglobin below 9 g/dL (OR = 3.284, *p* < 0.001), especially in severe anemia (hemoglobin <6 g/dL, OR 7.714, *p* < 0.001) (Table 4 and Figure 3).

**Table 4:** Associations between the hemoglobin level with adverse outcome in the AECOPD inpatients by multivariable logistic regression.

Hemoglobin level	Adverse outcome	
	OR (95% CI)	*P*-value
<6 g/dl	7.714(2.622 ~ 20.887)	**<0.001**
6 to <9 g/dl	3.284(2.142 ~ 4.93)	**<0.001**
9 to <12 g/dl	1.119(0.843 ~ 1.475)	0.432
12 to15.9 g/dl	reference	
16 to <18 g/dL	1.074 (0.602 ~ 1.789)	0.798
18 to <20 g/dl	1.824(0.731 ~ 3.914)	0.155
≥20 g/dl	2.282(0.352 ~ 8.336)	0.283

*P*-value < 0.05 were highlighted using the bold font.

## Discussion

This large multicenter cohort study found that severe AECOPD patients with anemia had higher risk of adverse outcomes during hospitalization, including in-hospital mortality, ICU admission, and invasive mechanical ventilation, than those with normal hemoglobin. Further stratified analysis showed that moderate and severe anemia patients had significantly higher odds ratios for adverse outcomes, while polycythemia did not exhibit this trend. This study for the first time to explore the relationship between hemoglobin levels and the risk of adverse outcomes during hospitalization for severe AECOPD.

Data from a large cohort (COPDGene cohort) indicated 9.2% of men and 3.5% of women had secondary polycythemia (21), but anemia is more frequently in people with COPD, with a reported prevalence of 7.5 to 34% (22). We found that 24.91% of the subjects with AECOPD had anemia, 6.39% of the subjects with AECOPD had polycythemia, similar to Nielsen’s found (35.2% of AECOPD patients had anemia and 6.8% had polycythemia) (23).

Polycythemia was associated with higher incidence of pulmonary embolism in COPD, and polycythemia in COPD patients was associated with more frequent re-hospitalization per year, more frequent rate of arrhythmia, a longer hospital stay and increased rate of mechanical ventilation (including noninvasive) and mortality, compared with COPD patients without polycythemia (15, 24). However, their studies main points to note polycythemia and long-term outcomes of COPD, the impact of polycythemia on the short-term outcomes of AECOPD has not been extensively explored. Recently, Nielsen et al. (23) revealed that readmission or mortality in AECOPD with polycythemia did not differ from the subjects with normal hemoglobin. However, the inclusion criteria for their study required subjects to be admitted with AECOPD as the primary diagnosis, or respiratory failure or pneumonia as the primary diagnosis alongside COPD as a secondary diagnosis. Our research, only enrolled severe AECOPD, still found that polycythemia does not significantly increase the risk of adverse outcomes (in-hospital mortality, invasive ventilation, ICU admission) when compared with normal hemoglobin. In fact, the SPIROMICS study (25) has pointed that polycythemia was associated with lower incidence of severe AECOPD than normal hemoglobin. The adverse outcomes of AECOPD may stem from uncorrected hypoxemia, a known predictor of mortality in COPD and long-term oxygen therapy (LTOT) has been proved can mitigate hypoxemia, reducing adverse outcomes (26, 27). Furthermore, in our study, patients with polycythemia were younger, had a better nutritional status (higher BMI and albumin), lower inflammatory markers (CRP, PCT, G test, GM test), which might also be one of the reasons why they had a lower risk of adverse outcomes. Hardly any published articles have confirmed that polycythemia is associated with the low-inflammatory state of COPD, further study is need to explore this phenomenon.

Several studies have confirmed that anemia is significantly associated with COPD exacerbations and reduced quality of life, hospitalization costs and duration, readmission, as well as mortality (18, 23, 28–30). Similar to the previous research results, our study found that anemia increases the risk of adverse outcomes among in-hospital severe AECOPD when compared to the normal hemoglobin. Previous studies have focused on COPD exacerbations, readmission and long-term prognosis, but adverse outcomes during hospitalization, which determine whether patients can recover and be discharged, have received little attention. For the first time, we explored the relationship between different hemoglobin stratification in hospitalized severe AECOPD and adverse clinical outcomes, when hemoglobin< 9 g/dL, the risk of adverse events significantly increases. In our study, anemia patients are more likely to have a higher proportion of comorbidities such as coronary heart disease and diabetes and diabetes, these diseases may deteriorate simultaneously during the COPD exacerbation and can also lead to a certain risk of adverse outcomes. Then, patients with anemia are older, and have lower BMI, worse albumin levels, renal function, and blood pressure, in other words, they have a greater risk of malnutrition and frailty, which may explain their higher risk of infection, for they have higher GM or G test positivity rates, and elevated inflammatory markers (CRP, PCT, WBC). Markoulaki et al. (31) also confirmed a negative association between hemoglobin and interleukin-6 (IL-6) during acute phase of COPD exacerbation, the other studies also observed lower hemoglobin levels alongside higher CRP in AECOPD patients (17, 32). Anemia caused by inflammation, infection and malnutrition forms a vicious cycle, which aggravates each other and eventually leads to worse adverse outcomes.

In fact, systemic inflammation plays a key role in anemia development among COPD patients (33–35). These patients demonstrate elevated inflammatory markers including CRP, tumor necrosis factor alpha (TNF-α), and IL-6. CRP production (primarily regulated by IL-6 and indirectly by TNF-α) and TNF-α’s direct suppression of erythrocyte progenitor cell proliferation both contribute to anemia. Additionally, IL-6 upregulates hepcidin, impairing iron absorption and release, which leads to functional iron deficiency and further exacerbates anemia (34, 36, 37). Elevated PCT typically suggests bacterial infection, which may cause hemolytic anemia through toxin production, immune activation, or direct erythrocyte damage. COPD patients also frequently exhibit nutritional deficiencies (particularly albumin, iron, vitamin B12, and folate), further predisposing to anemia (35). Ultimately, impaired erythropoiesis, reduced erythropoietin production, shortened red blood cell survival, and iron dysregulation collectively cause anemia (38–40). Markoulaki et al. (31) found when AECOPD transitions to resolution and stable phases, the hemoglobin level rises while the inflammatory indicators also decline significantly. Our study comparing hemoglobin levels found that anemia, particularly moderate-to-severe cases, correlated with worse outcomes during hospitalization, that suggested clinicians the importance of correcting anemia in AECOPD patients and breaking the vicious cycle between anemia, inflammation, and malnutrition to reduce hospitalization costs and duration, and lower the risk of adverse events and ultimately improve the prognosis of patients.

This study has several strengths. First, as a multicenter, large-scale real-world study, it consecutively enrolled unselected hospitalized AECOPD patients and comprehensively collected data including demographics, comorbidities, and laboratory results. Second, while previous research primarily focused on stable COPD, we specifically examined acute exacerbations, which impose greater economic and health burdens. Finally, we performed multiple hemoglobin subgroup analyses to confirm the association between moderate-to-severe anemia and adverse outcomes. However, some limitations exist. The study lacked serum iron and ferritin measurements, preventing assessment of iron-deficiency anemia prevalence. Additionally, without serial hemoglobin data, we could not evaluate whether anemia correction improves prognosis. Finally, this study only included people with severe AECOPD and lacked data on those with mild and moderate AECOPD, we failed to analyze whether the clinical outcomes of all AECOPD patients were affected by anemia.

In conclusion, anemia (particularly <9 g/dL,) on-admission, not polycythemia, was associated with an increased risk of adverse outcomes, which may serve as an effective biomarker of poor short-term prognosis among inpatients with severe AECOPD. This finding may remind the clinicians should attach more importance to further exploration and correction of the causes of anemia and correcting anemia itself in patients with AECOPD.

## Data Availability

The original contributions presented in the study are included in the article/Supplementary material, further inquiries can be directed to the corresponding authors.
