# Supplementary material for: Hemoglobin and clinical outcomes of in-hospital patients with severe acute exacerbation of chronic obstructive pulmonary disease: a multicenter cohort study
Source: Front Med (Lausanne). 2025 Oct 16;12:1674268. doi: 10.3389/fmed.2025.1674268 (PMC12571645; doi:10.3389/fmed.2025.1674268)
Supplement: Supplementary file 1 [file Table_1.docx]

**Supplemental table 1** Baseline Characteristics and laboratory tests of the Study Population

| **Variables** | **Total population**  **（N=9660）** | | | | **Non-adverse outcomes**  **（N=9328）** | | **Adverse outcomes**  **(N=332)** | ***p* value** |
| --- | --- | --- | --- | --- | --- | --- | --- | --- |
| **Characteristics** | | | | | | | | |
| Female | 1,888 (19.54) | | | | 1,815 (19.46) | | 73 (21.99) | 0.284 |
| Age(years) | 71.68 ± 9.86 | | | | 71.60 ± 9.85 | | 74.20 ± 9.65 | **<0.001** |
|  |  | | | |  | |  |  |
| Minority | 259 (2.68) | | | | 254 (2.72) | | 5 (1.51) | 0.240 |
| BMI (kg/m^2^) | 21.56 ± 3.02 | | | | 21.56 ± 3.02 | | 21.45 ± 3.02 | 0.511 |
| Smoking history |  | | | |  | |  | **0.004** |
| Never | 3,568 (36.94) | | | | 3,423 (36.70) | | 145 (43.67) |  |
| Current smoker | 1,936 (20.04) | | | | 1,890 (20.26) | | 46 (13.86) |  |
| Former smoker | 4,156 (43.02) | | | | 4,015 (43.04) | | 141 (42.47) |  |
| **Comorbidities** | | | | | | | | |
| Hypertension | 3,103 (32.12) | | | | 2,977 (31.91) | | 126 (37.95) | **0.024** |
| Coronary heart disease | | | | 982 (10.17) | 935 (10.02) | | 47 (14.16) | **0.018** |
| Chronic heart failure | | 781 (8.08) | | | 709 (7.60) | | 72 (21.69) | **<0.001** |
| Cor pulmonale | | 1,669 (17.28) | | | 1,563 (16.76) | | 106 (31.93) | **<0.001** |
| Diabetes | 1,074 (11.12) | | | | 1,030 (11.04) | | 44 (13.25) | 0.242 |
| OSA | 58 (0.60) | | | | 56 (0.60) | | 2 (0.60) | 1.00 |
| **Symptoms and signs** | | | | | | | | |
| SBP (mmHg) | 132.35 ± 18.95 | | | | 132.38 ± 18.82 | | 131.54 ± 22.32 | 0.430 |
| DBP (mmHg) | 79.53 ± 12.17 | | | | 79.62 ± 12.07 | | 76.94 ± 14.46 | **<0.001** |
| Pulse (times/min) | 88.97 ± 16.49 | | | | 88.91 ± 16.29 | | 90.67 ± 21.32 | 0.056 |
| Respiratory rate (times/min) | 20.82 ± 2.06 | | | | 20.82 ± 1.92 | | 20.88 ± 4.39 | 0.579 |
| **Laboratory Tests** | | | | | | | | |
| Hemoglobin (g/dl) | | |  | | |  |  | **<0.001** |
| Normal | 6,637 (68.71) | | | | 6,451 (69.16) | | 186 (56.02) |  |
| Polycythemia | 617 (6.39) | | | | 596 (6.39) | | 21 (6.33) |  |
| Anemia | 2,406 (24.91) | | | | 2,281 (24.45) | | 125 (37.65) |  |
| WBC (10^9^/L) | 7.575(5.84,9.823) | | | | 7.555(5.83,9.79) | | 9.08(6.3,11.593) | **<0.001** |
| Neutrophil ratio (%) | | 74.1(64.6,83) | | | 73.8(64.3,82.525) | | 83.6(75.9,90.2) | **<0.001** |
| Eosinophil ratio (%) | 1.10(0.20,2.80) | | | | 1.20(0.20,2.80) | | 0.20(0,1.03) | **<0.001** |
| Platelet(10^9^/L) | 195(149,251) | | | | 196(150,252) | | 160.5  (122.75,220.25) | **<0.001** |
| PCT (mg/mL) | 0.05(0.05,0.06) | | | | 0.05(0.05,0.06) | | 0.0835  (0.05,0.243) | **<0.001** |
| CRP (mg/dl) | 11.1  (6.448,11.505) | | | | 11.1  (6.318,11.1) | | 12.6  (10.175,63.025) | **<0.001** |
| G test (%) | 173 (1.79) | | | | 163 (1.75) | | 10 (3.01) | 0.134 |
| GM test (%) | 117 (1.21) | | | | 98 (1.05) | | 19 (5.72) | **<0.001** |
| PH | 7.40 ± 0.04 | | | | 7.40 ± 0.04 | | 7.39 ± 0.06 | 0.08 |
| PaO_2_ (mmHg) | 82.7(73.6,97.2) | | | | 82.7(73.9,97.1) | | 82.7  (67.925,102.075) | 0.06 |
| PaCO_2_ (mmHg) | 45.54 ± 10.88 | | | | 45.45 ± 10.73 | | 48.10 ± 14.25 | **<0.001** |
| LAC (mmol/L) | 1.5(1.5,1.5) | | | | 1.5(1.5,1.5) | | 1.5(1.3,1.7) | 0.5 |
| Albumin (g/dl) | 37.12 ± 5.23 | | | | 37.20 ± 5.22 | | 34.78 ± 5.10 | **<0.001** |
| BUN (mmol/L) | 5.58(4.3,7.2) | | | | 5.52(4.26,7.1) | | 7.39  (5.58,11.063) | **<0.001** |
| Creatinine(μmol/L) | 75(62,92.725) | | | | 75(62.3,92.225) | | 75(58,105.45) | 0.9 |
| NT-proBNP (ng/dl) | 259.25  (95.908,557.4) | | | | 259.25  (93,514.475) | | 659.5  (259.25,2939.75) | **<0.001** |
| D-dimer (mg/dl FEU) | | 0.692(0.4,1.11) | | | 0.692(0.4,1.07) | | 1.03  (0.692,2.243) | **<0.001** |

**Notes:** Data are presented as the number of patients (%), mean ± standard deviation, median (interquartile range). Those with *P* value < 0.05 were highlighted using the bold font.

**Abbreviations:** BMI, body mass index. OSA, obstructive sleep apnea; SBP, systolic blood pressure; DBP, diastolic blood pressure;WBC, white blood cell; PCT, procalcitonin; CRP, C-reactive protein; G test, 1,3-β-D glucan test; GM test, galactomannan test; PH, potential of hydrogen; PaO_2,_ partial pressure of oxygen in arterial blood; PaCO_2_, partial pressure of carbon dioxide in arterial blood; LAC, lactic acid; BUN, blood urea nitrogen; NT-proBNP, N-terminal pro-brain natriuretic peptide.

**Supplemental table 2** Multivariate Analysis on Adverse Outcomes in Patients with AECOPD

| Variables | **OR** | **95%CI** | ***P* value** |
| --- | --- | --- | --- |
| Polycythemia | 0.954 | 0.574~1.51 | 0.847 |
| Anemia | 1.477 | 1.142~1.903 | **0.003** |
| Age(years) | 1.001 | 0.989~1.014 | 0.858 |
| Hypertension | 1.317 | 1.03~1.678 | **0.027** |
| Coronary heart disease | 1.073 | 0.75~1.504 | 0.693 |
| Chronic heart failure | 2.273 | 1.673~3.054 | **<0.001** |
| Cor pulmonale | 1.740 | 1.344~2.239 | **<0.001** |
| DBP | 0.987 | 0.978~0.996 | **0.004** |
| WBC | 1.031 | 1.006~1.056 | **0.013** |
| Neutrophil ratio | 1.043 | 1.029~1.058 | **<0.001** |
| Eosinophil ratio | 0.933 | 0.854~1.009 | 0.109 |
| Platelet | 0.996 | 0.994~0.997 | **<0.001** |
| Albumin | 0.946 | 0.924~0.969 | **<0.001** |
| BUN | 1.004 | 1.001~1.008 | **0.012** |
| NT-proBNP (ng/dl) | 1.000 | 1~1 | 0.743 |
| D-dimer | 1.001 | 0.99~1.005 | 0.734 |
| PCT | 1.013 | 1.001~1.023 | **0.016** |
| CRP | 1.003 | 1.002~1.005 | **<0.001** |

Those with *P* value < 0.05 were highlighted using the bold font.

**Abbreviations:** DBP, diastolic blood pressure; WBC, white blood cell; PCT, procalcitonin; CRP, C-reactive protein; BUN, blood urea nitrogen; NT-proBNP, N-terminal pro-brain natriuretic peptide.
